# Psoriasis and Microbiota: A Systematic Review

**DOI:** 10.3390/diseases6020047

**Published:** 2018-06-02

**Authors:** Farida Benhadou, Dillon Mintoff, Benjamin Schnebert, Hok Bing Thio

**Affiliations:** 1 Dermatology Department, Erasme Hospital, Université Libre de Bruxelles, Route de Lennik 808, Anderlecht, 1070 Brussels, Belgium; 2 Dermatology Department, Sir Paul Boffa Hospital, 1491 Floriana, Malta; dillon.mintoff@gov.mt; 3 Medecine Faculty, Université Libre de Bruxelles, 1070 Brussels, Belgium; bschnebe@ulb.ac.be; 4 Dermatology Department, Erasmus Medical Center, 3000 Rotterdam, The Netherlands; h.thio@erasmusmc.nl

**Keywords:** psoriasis, microbiota, probiotics, prebiotics

## Abstract

Background: Recent advances have highlighted the crucial role of microbiota in the pathophysiology of chronic inflammatory diseases as well as its impact on the efficacy of therapeutic agents. Psoriasis is a chronic, multifactorial inflammatory skin disorder, which has a microbiota distinct from healthy, unaffected skin. Aim: Through an extensive review of the literature, we aim to discuss the skin and gut microbiota and redefine their role in the pathogenesis of psoriasis. Conclusions: Unfortunately, the direct link between the skin microbiota and the pathogenesis of psoriasis remains to be clearly established. Apart from improving the course of psoriasis, selective modulation of the microbiota may increase the efficacy of medical treatments as well as attenuate their side effects.

## 1. Introduction

Even though psoriasis is one of the most studied immune-mediated inflammatory skin diseases, its pathogenesis is still not yet fully understood [1,2]. Psoriasis is the result of interplay between predisposing genetic factors and the influence of external triggers [3]. The commensal cutaneous microbiota plays a crucial role in maintaining skin integrity and function as a critically exposed barrier [4]. Research has shown that the composition of the cutaneous microbiota is related to many dermatological diseases including, but not limited to, psoriasis, atopic dermatitis, and acne vulgaris [5,6].

The correlation of gut microbiota with immune-mediated diseases has shone a spotlight on the pathogenic role of the microbiota. Profiling studies have recently identified characteristic shifts in the gut microbiota, which have been associated with the pathogenesis of inflammatory bowel diseases (IBD), reinforcing the hypothesis that IBD results from altered interplay between gut microbes and the mucosal immune system [7,8]. Interestingly, the intestinal microbiota is able to affect extracolonic sites, such as the skin, joints, lungs, liver, and nervous and cardiovascular systems.

The clinical significance of the interaction between microbiota and the immune system is of major influence and it can provide new insights into the pathogenesis of chronic inflammatory skin diseases and allow for the development of microbiota-targeted therapeutic options. This review will shift the spotlight onto the microbiotas’ association with skin disease, with a particular focus on psoriasis.

## 2. Composition of the Healthy Human Microbiota of the Skin and Gut

The close relationship between skin and microbiota has been systemically evaluated. Microscopic bacteria, fungi, and viruses inhabit the harsh, acidic environment of the skin. The inhomogeneity of the healthy microbiota is not only physical (with a distinct microbiota between dry, moist, and apocrine rich skin), but also temporal (with some organisms considered resident and others considered transient) [8]. The human microbiota plays a key role in various aspects of disease, ranging from its pathogenesis to treatment response.

The accessibility of non-culture-dependent techniques such as DNA and ribosomal RNA sequencing (16S and 18S) have facilitated the discovery, classification, and association of specific types of human flora components with specific diseases [9]. In general terms, four bacterial phyla dominate the healthy skin microbiota, namely Actinobacteria, Firmicutes, Proteobacteria, and Bacteroidetes, of which the genera *Corynebacterium*, *Propionibacterium*, and *Staphylococcus* are most abundant [8]. Host-dependent factors that have a direct influence on the variation of the skin microbiota include its physiology, external environment, immune system, lifestyle, and underlying medical conditions [10].

The number of organisms composing the gut microbiota exceed the total human-host cell population ten-fold [11]. This organ within an organ, like skin, is characterized by a diverse range of microorganisms in the healthy state [12,13].

## 3. Dysbiosis of the Skin Microbiota

The intricate relationship between the microbiota and host begins at birth, as pelvic floor commensals are transmitted vertically during parturition and during breastfeeding via microbe-rich colostrum. An immature neonatal immune system which is yet unable to have a full immune response is thought to allow for a symbiotic relationship between microbiota. This is evidenced by the different way in which neonatal innate cells respond to microbial ligands, having an attenuated inflammatory response when compared to adult cells [14].

Communication between the host and commensal is made possible through microbial-associated molecular patterns (MAMPs). The conventional wisdom that the immune system recognizes foreign from innate and subsequently activates a targeted response to nonself is challenged, as no immune response is brought about to commensal skin and mucosal microbiota MAMPs, despite the continuous interaction of MAMPs with specific pattern recognition receptors (PRRs), which are traditionally known to recognize microbial ligands and initiate an immune response [15]. 

Through such communication, the commensal microbiota can influence the development of the hosts’ postnatal immune system [14].

Additional physical barriers, such as the epithelial cell layer (with its continuous secretion of IgA) and Goblet cells producing mucus, isolate the microbiota from a direct contact with the mucosal layer, but at the same time allow it to exert local and system effects [16].

Dysbiosis has become a key research focus in the pathophysiology of microbiota-associated chronic inflammatory diseases such as inflammatory bowel disease [17]. Described as an imbalance between the microbiota and its host, dysbiosis can be considered a form of impaired homeostasis in which the microbiota is shifted towards a less complex, less varied pathological spectrum [18]. The trigger that causes the shift from symbiosis to dysbiosis is not yet fully elucidated, however, a genetic basis has been suggested [17].

Described as the imbalance between damaging oxidative free radicals and protective antioxidants [19], oxidative stress is a repercussion of gut dysbiosis and a mechanism which predisposes to pathology. Reactive oxygen species (ROS) have been identified as a link between an altered microbiota and disease. It has been suggested that a dysbiotic microbiota increases the production of nitric oxide (NO) and nitric oxide synthases (NOS) as an inflammatory response, which subsequently impair DNA repair systems and cause cell membrane dysfunction [18]. This is demonstrated by the production of the effector protein EspF by enteropathogenic *Escherichia coli*, resulting in the downregulation of host DNA mismatch repair proteins, predisposing to carcinogenesis [20].

Thus, understanding the implications of dysbiosis on disease can lead to the development of novel targeted therapies.

## 4. Microbiota and Psoriasis

The study of the role of microorganisms in the pathophysiology of skin disease dates back to the first half of the 20th century, where a possible link between commensal nasopharyngeal streptococci and psoriasis was investigated [21,22]. Concrete evidence linking both conditions failed to be established at the time; however, later work by Norrlind et al. successfully correlated streptococcal pharyngitis (as evidenced by pharyngeal swab culturing and serology) with guttate psoriasis as well as exacerbations of stable chronic plaque psoriasis [23,24].

### 4.1. Bacteria’s Role in Psoriasis

Equipped with the knowledge of the role of T cells in the aetiology of psoriasis, the missing link between streptococcal infection and unstable/guttate psoriasis was sought [25]. M proteins, found on Group A, C, and G β-haemolytic streptococci, were implicated, as the worsening of chronic plaque psoriasis was only associated with these M-protein-yielding bacteria [26]. It is postulated that M proteins may be mimicking keratin determinants with subsequent psoriatic T-cell activation [26,27]. This theory is substantiated by the fact that the interaction between type IV collagen and α1β1 integrin found exclusively on epidermal psoriatic T cells results in the expansion of this subset of cells and the eventual manifestation of psoriasis [28]. T-cell activation in guttate psoriasis is also under the influence of antigens such as streptococcal pyogenic toxin A and B as well as peptidoglycan [29,30,31].

It seems clear that the skin microbiota may have a role in the pathogenesis of chronic plaque psoriasis. *Corynebacterium*, *Propionibacterium*, *Staphylococcus*, and *Streptococcus* have been identified as the major bacterial genera. Different “cutaneo-types” such as “Proteobacteria-associated”, “Firmicutes-associated”, and “Actinobacteria-associated” are the most prevalent [32]. Using non-culture techniques, Firmicutes was found to be the most abundant phylum of bacteria in lesional psoriatic skin, whereas Actinobacteria was significantly underrepresented in psoriatic skin lesions when compared to healthy and nonlesional skin [33,34].

### 4.2. Fungi’s Role in Psoriasis

Fungi have shared the spotlight with bacteria in the possible microbiological association between psoriasis and the microbiota. In the 1980s, suspensions of *Malassezia ovalis* fragments applied to the unaffected skin of patients suffering from psoriasis induced the formation of psoriatic plaques in all 10 tested subjects [35]. Such psoriasiform reactions to *Malassezia* were also observed after the indirect deposition of the fungus from the scalp onto skin of patients with psoriasis. In this case series, psoriatic lesions responded to a one-week course of oral antifungal therapy [36]. An increased concentration of *Malassezia* yeast in lesional skin has also been associated with exacerbations of psoriasis [37].

*Malassezia*’s role in psoriasis may be due to its ability to upregulate the expression of tumour growth factor-β1, integrin chain, and HSP70, thereby promoting immune cell migration and keratinocyte hyperproliferation in patients with psoriasis [38,39]. The humoral response of patients with psoriasis to *Malassezia furfur* is associated with higher IgG and lower IgM when compared to healthy individuals [40,41].

As with the bacterial microbiota, non-culture methods have superseded culture methods for the detection of *Malassezia* patterns in psoriatic and non-psoriatic skin [42,43,44,45,46]. Culture- and non-culture-based research has produced congruent results, with *M. restricta* being the most commonly identified *Malassezia* species.

Despite the constant findings, identification of the fungus cannot be used as a marker to distinguish psoriatic from non affected skin and psoriatic from healthy skin.

## 5. The Impact of the Skin Microbiota and Comorbidities

Psoriatic arthritis affects up to 30% of patients with psoriasis, causing considerable morbidity from symptoms directly related to the condition as well as comorbid diseases, such as cardiovascular disease, which is more prevalent in patients with psoriatic arthritis [47,48]. Despite the likely association between psoriatic arthritis and the skin microbiota, there is currently no evidence establishing a direct link between both conditions [49].

Fungi are also implicated in the pathophysiology of comorbid nail psoriasis, which is more prevalent in patients with psoriatic arthritis [49]. The association was made after clinical improvement of nail psoriasis was noted after affected patients were treated with an oral antifungal [50].

Currently, most research is directed toward the role of the gut (rather than skin) microbiota in the pathogenesis of inflammatory arthritis [51].

## 6. Skin Microbiota Regulates Treatment Efficacy

### 6.1. Conventional Therapeutic Options for Psoriasis May Modulate the Composition of Skin Microbiota

If the skin microbiota has a role in the pathogenesis of psoriasis, then one would be inclined to ask whether it could be manipulated for therapeutic intent and whether its resilience has ramifications on the effectiveness of psoriatic treatment.

Martin et al. demonstrated that clinical improvement of psoriatic plaques after balneotherapy is associated with an alteration of lesional skin microbiota such that it becomes more closely resembling that of unaffected skin after therapy [52].

Furthermore, a recent study by Darlenski et al. provides insights into how improvement in psoriatic plaques after treatment with narrow-band ultraviolet radiation (NB-UVB), a conventional systemic therapy for the treatment of psoriasis, is associated with an improvement into the previously alluded systemic oxidative stress parameter ROS as well as others, such as malondialdehyde (MDA) and ascorbyl radicals. The authors conclude that the improvement in oxidative stress parameters is a direct reflection of improvement in disease activity [19]. Treatment with NB-UVB has already been shown to cause significant alterations in the skin microbiota [53]. It would be interesting to investigate the relationship between clinical improvement of psoriasis, alteration in skin microbiota, and systemic oxidative stress parameters after NB-UVB therapy.

### 6.2. Modifying the Gut Microbiota as Therapeutic Option in Psoriasis

The manipulation of the gut microbiota either by its selective augmentation through the introduction of live organisms (probiotic) or by skewing growth positively through the administration of non digestible carbohydrates (prebiotic) has long been established.

Promising results have been achieved with the use of pre- and probiotics in atopic dermatitis, acne, and wound healing [54]. Pre- and probiotics exert immunomodulatory effects on skin and can strengthen its barrier function by decreasing skin bacterial load as well as opposing aggressive commensals [54,55]. Such results further consolidate the idea of a direct link between gut and skin microbiota and imply that an “artificial” microbiota can modulate the “natural” microbiota for therapeutic purposes. Research supporting such a claim comes from anecdotal evidence documenting positive therapeutic response of pustular psoriasis to the administration of the probiotic *Lactobacillus* [55].

### 6.3. Interaction of Microbiota with Treatment

The skin microbiota also interacts at a local level with topical treatment in psoriasis. It has been demonstrated that a psoriatic skin associated with a high concentration on *Malassezia* spp. is significantly more likely to become irritated when treated with topical calcipotriol, a vitamin D analogue used as a first-line topical treatment in psoriasis [56,57]. In the same study, facial and scalp skin irritation was significantly lower in patients simultaneously treated with oral itraconazole; however, this did not improve treatment efficacy.

Topical calcipotriol may in and of itself influence skin *Malassezia* population by upregulating cathelicidin, an antimicrobiobal peptide having activity against the fungus [58,59].

More research is required to demonstrate whether established treatment for psoriasis and the skin microbiota could be harmonized to achieve a better clinical response and if directly altering the microbiota (as a novel therapeutic target) to make it fit with the normal “cutaneo-type” as much as possible has any therapeutic implications [33]. 

## 7. Diet and Microbiota: What Can We Learn from Psoriasis?

The gut and skin are intricately related through what is referred to as the “gut–skin axis” [60]. A vast microbiota in excess of 10^14^ microbes saturates the convoluted lumen of the human gut [61]. Apart from its symbiotic relationship with the gut on a local level, this organ within an organ also exerts systemic effects on the rest of the body, including the skin [62]. 

In the setting of psoriasis, the gut microbiota seems to be considerably altered, with a significantly reduced abundance of *Akkermansia muciniphilia* when compared to controls [63].

When it comes to the association between the gut and skin, the role of *Lactobacillus* has been targeted. Through a randomized, double-blind, placebo-control study, Gueniche et al. observed that patients who are administered a daily oral dose of *Lactobacillus paracasei* NCC2461 exhibit decreased skin sensitivity, have a hastened barrier function recovery, and are more efficient in preserving the skin moisturizing agents urea and sodium lactate [64].

Interestingly, Chen et al. demonstrated that mice fed *Lactobacillus pentosus* developed a milder form of imiquimod-induced psoriasis when compared to mice fed with a vehicle control [65]. Moreover, it has been shown that mice fed with *Lactobacillus pentosus* have suppressed psoriasis-related pro-inflammatory and Th17-associated cytokines, such as TNF-α, IL-6, IL-23, IL-17A, IL-17F, and IL-22 [66]. The direct role of *Lactobacillus* in human patients with psoriasis still needs to be investigated.

Such evidence is of major clinical relevance as it helps to understand how to tip the “microbiota balance” positively. In the context of psoriasis, the role of diet (often coupled with exercise) has often been promoted claimed in for its capacity to modulate and improve patients’ psoriatic plaques and treatment efficacy [67,68].

Prompted by the discovery of significantly higher mean IgA anti-gliadin antibodies (AGA) in a cohort of 302 patients with psoriasis when compared to a reference group, Michaëlsson et al. set out to evaluate the response of disease to a gluten-free diet in patients with positive AGA tests [69,70].

Psoriasis skin improvement has been assessed by calculating the clinical Psoriasis Area Severity Index (PASI). PASI evaluates the severity of erythema, scaling, induration, and skin surface extension. The IgA-AGA-positive group of patients adhering to a gluten-free diet were noted to have a highly significant reduction in PASI when compared to the IgA-AGA-negative group. Of note, 60% of IgA-AGA-positive patients experienced a deterioration in their skin condition when they reintroduced their habitual diet. None of the IgA-AGA-negative patients noted any changes in their skin condition after going back to their habitual diet [70,71]. After such encouraging results linking diet and skin in a subset of patients with psoriasis, the author subsequently set out to establish histological skin changes in psoriatic and unaffected skin of patients with psoriasis with or with positive IgA and/or IgG AGA on a gluten-free diet. After adhering to a gluten-free diet for three months, patients were noted to have a significantly decreased dermal Ki-67+ cell population (an indicator of cell proliferation) in lesional skin. The reduction of the Ki-67+ cell population in unaffected skin was statistically significant in the dermis, whilst in epidermal regions, a gluten-free diet led to a reduction in Ki-67 positivity, albeit to a statistically insignificant extent. The areas of psoriatic skin expressing Ki-67 did not regress with a gluten-free diet. Dermal tissue transglutaminase, notably more concentrated (8×) in lesional skin when compared to uninvolved skin, was also noted to decrease by half after the adoption of a gluten-free diet in AGA-positive patients. Adhering to a gluten-free diet also led to a significant decrease in the lesional CD4+ T-lymphocyte count of AGA-positive patients. No significant changes were noted in the skin of AGA-negative patients after treatment with a gluten-free diet [71].

Interestingly, the gut microbiota of patients with psoriasis was shown to have a higher Firmicutes/*Bacteroides* ratio when compared to matched healthy controls (9.02 vs 3.18, *p* < 0.001) A significant positive correlation was also established between the Firmicutes/*Bacteroides* ratio and mean PASI, suggesting that a shift in gut microbiology towards a more inflammatory subset (Firmicutes-dominant) might be part of the aetiopathogy of psoriasis. Of note, Actinobacteria counts are also noted to be significantly higher in the unaffected individuals when compared to the patients with psoriasis group [72].

## 8. Conclusions

Psoriasis is a common skin inflammatory condition, affecting approximately 3% of the worldwide population and resulting from a combination of genetic and environmental factors. Ecological diversity of the microbial population overlaying the lesions is greater than that of healthy skin. It has yet to be established whether such alterations in the microbiota are a cause or a consequence of disease. Interactions between microbiota and the immune system are important for establishing and maintaining host homeostasis. Modification of the composition of microbiota could lead to a shift in immune system activation and eventually to the development of inflammatory diseases.

Emerging evidence supports the existence of a dynamic interplay and communication axes between organs, such as the gut–skin axis.

The dysregulated skin microbiota may become a novel therapeutic target in psoriatic patients. Restoration of symbiosis may also increase the efficacy of already established medical treatments. For these reasons, further research into the selective modulation of skin microbiota is required.
